# P-2180. Evaluation of Cytomegalovirus Resistance at an Academic Medical Center

**DOI:** 10.1093/ofid/ofaf695.2343

**Published:** 2026-01-11

**Authors:** Jeffrey C Pearson, Lindsey R Baden, Lisa A Cosimi, Benjamin Gewurz, Eric M Gillett, Simran Gupta, Nicolas C Issa, David W Kubiak, Alexis Liakos, Jessica S Little, Pritha Sen, Ann E Woolley

**Affiliations:** Brigham and Women's Hospital, Boston, MA; Brigham and Women's Hospital, Boston, MA; Brigham & Women's Hospital, Boston, Massachusetts; Brigham & Women's Hospital, Harvard Medical School, Boston, Massachusetts; Brigham and Women's Hospital, Boston, MA; Brigham and Women's Hospital, Boston, MA; Brigham & Women's Hospital, Boston, Massachusetts; Brigham & Women's Hospital, Boston, Massachusetts; Brigham & Women's Hospital/Dana-Farber Cancer Institute, boston, Massachusetts; Brigham and Women's Hospital, Boston, MA; Brigham and Women's Hospital, Boston, MA; Brigham and Women's Hospital, Boston, MA

## Abstract

**Background:**

Cytomegalovirus (CMV) infection causes significant morbidity and mortality in immunocompromised patients. Despite novel antiviral agents like maribavir and letermovir, resistance remains a concern. This study describes CMV resistance rates at a large academic medical center.

**Methods:**

Patients tested for CMV antiviral resistance from January 2022 through March 2025 were included, while tests with insufficient sample volume were excluded. The study's primary outcome was the prevalence of CMV resistance to various antivirals in patients who had testing completed. Secondary outcomes included the most encountered resistance mutations. All data was analyzed using descriptive statistics. This study was deemed exempt by the Mass General Brigham Institutional Review Board (protocol 2025P001067).

**Results:**

From January 2022 through March 2025, CMV resistance testing was performed 123 times in 76 unique patients. Insufficient serum quantities were collected in 47 tests ordered, so the analysis included 76 tests from 53 unique patients. Resistance was detected overall in 29/76 tests (38.2%). UL97 phosphotransferase mutations conferring resistance to ganciclovir were identified in 23/66 tests (34.8%), most commonly A594V and C603W. UL97 phosphotransferase mutations conferring resistance to maribavir were identified in 9/61 tests (14.8%), most commonly H411Y and T409M. UL54 DNA polymerase mutations potentially conferring resistance to ganciclovir, foscarnet, and cidofovir were identified in 2/69 tests (2.9%). UL56 terminase mutations conferring resistance to letermovir were identified in 2/9 tests (22.2%). When UL97 mutations conferring resistance to ganciclovir were identified, maribavir resistance was identified in 5 of 21 tests (23.8%), and all nine tests that resulted in maribavir resistance occurred in patients previously exposed to maribavir.

**Conclusion:**

In our cohort, CMV resistance to ganciclovir was most frequently observed. Though less common, resistance to novel agents like maribavir and letermovir was identified in patients previously exposed to these antivirals. The development of resistance is correlated with increased morbidity and mortality, so timely resistance testing is paramount to help guide management and improve outcomes.

**Disclosures:**

Jeffrey C. Pearson, PharmD, InflaRx Pharmaceuticals, Inc.: Advisor/Consultant Lindsey R. Baden, MD, Bill & Melinda Gates Foundation: Grant/Research Support|Bill & Melinda Gates Foundation: Collaboration of clinical trials conducted|COVID Vaccine Prevention Network: Collaboration of clinical trials conducted|Food and Drug Administration: Advisor/Consultant|HIV Vaccine Trials Network: Collaboration of clinical trials conducted|International AIDS Vaccine Initiative: Collaboration of clinical trials conducted|Johnson & Johnson: Collaboration of clinical trials conducted|Military HIV Research Program: Collaboration of clinical trials conducted|Moderna, Inc.: Collaboration of clinical trials conducted|National Institutes of Health: Advisor/Consultant|National Institutes of Health: Grant/Research Support|National Institutes of Health: Collaboration of clinical trials conducted|The Ragon Institute: Collaboration of clinical trials conducted|Wellcome Trust: Grant/Research Support Benjamin Gewurz, MD, PhD, Alnylam: Stocks/Bonds (Public Company)|fennec: Stocks/Bonds (Public Company)|moderna: Stocks/Bonds (Public Company)|passage: Stocks/Bonds (Public Company)|Regeneron: Stocks/Bonds (Public Company)|UpToDate: Advisor/Consultant|UpToDate: receive small stipend for serving as a topic reviewer|Vertex Pharmaceuticals: Stocks/Bonds (Public Company) Nicolas C. Issa, MD, AiCuris: Grant/Research Support|Astellas: Grant/Research Support|Boehringer Inglheim: Advisor/Consultant|Fujifilm: Grant/Research Support|GSK: Grant/Research Support|Merck: Grant/Research Support|Moderna: Grant/Research Support David W. Kubiak, PharmD, BCPS, BCIDP, FIDSA, Astellas Pharma US, Inc.: Advisor/Consultant Jessica S. Little, MD, Merck: Grant/Research Support|Moderna, Inc.: Collaborative research

